# Host Responses to *Malassezia* spp. in the Mammalian Skin

**DOI:** 10.3389/fimmu.2017.01614

**Published:** 2017-11-22

**Authors:** Florian Sparber, Salomé LeibundGut-Landmann

**Affiliations:** ^1^Section of Immunology, Vetsuisse Faculty, University of Zürich, Zürich, Switzerland

**Keywords:** *Malassezia*, commensalism, opportunistic pathogenic fungi, skin disorders, innate immunity, adaptive immunity, allergic response, indoles

## Abstract

The skin of mammalian organisms is home for a myriad of microbes. Many of these commensals are thought to have beneficial effects on the host by critically contributing to immune homeostasis. Consequently, dysbiosis can have detrimental effects for the host that may manifest with inflammatory diseases at the barrier tissue. Besides bacteria, fungi make an important contribution to the microbiota and among these, the yeast *Malassezia* widely dominates in most areas of the skin in healthy individuals. There is accumulating evidence that *Malassezia* spp. are involved in a variety of skin disorders in humans ranging from non- or mildly inflammatory conditions such as dandruff and pityriasis versicolor to more severe inflammatory skin diseases like seborrheic eczema and atopic dermatitis. In addition, *Malassezia* is strongly linked to the development of dermatitis and otitis externa in dogs. However, the association of *Malassezia* spp. with such diseases remains poorly characterized. Until now, studies on the fungus–host interaction remain sparse and they are mostly limited to experiments with isolated host cells *in vitro*. They suggest a multifaceted crosstalk of *Malassezia* spp. with the skin by direct activation of the host *via* conserved pattern recognition receptors and indirectly *via* the release of fungus-derived metabolites that can modulate the function of hematopoietic and/or non-hematopoietic cells in the barrier tissue. In this review, we discuss our current understanding of the host response to *Malassezia* spp. in the mammalian skin.

## Introduction

*Malassezia* spp. are lipophilic yeasts, which are part of the skin microbiota of many mammals and birds. In fact, the genus *Malassezia* is by far the most abundant eukaryotic member of the microbial flora of the skin in these organisms (1). Most *Malassezia* spp. have a predilection for seborrheic skin sites such as the scalp and the trunk. They rely on exogenous fatty acid sources for their nutritive requirements because of their lack of genes encoding for the fatty acid synthase and genes involved in carbohydrate metabolism (2–4). In agreement, the cell wall of *Malassezia* spp. is particularly rich in lipids (5).

The genus *Malassezia* currently comprises 17 species, three of which have only recently been proposed (6–8). *Malassezia globosa, Malassezia restricta*, and *Malassezia sympodialis* are most frequently isolated from the healthy human skin with distinct relative frequencies at specific body sites (1, 9). The age of the host and geographic factors also influence their distribution (10). *Malassezia pachydermatis, Malassezia nana*, and *Malassezia caprae* are found predominantly in non-human hosts (6). Surprisingly, the microbial communities of the skin are astonishingly stable and maintained over time, despite the skin’s exposure to the external environment (11). It is currently unknown whether *Malassezia* spp. play a mutualistic role and may thus contribute to immune homeostasis of the host.

Apart from their commensal nature, *Malassezia* spp. are also associated with common skin disorders such as pityriasis versicolor and seborrheic dermatitis as well as more severe inflammatory skin pathologies including atopic eczema and atopic dermatitis in humans (10) and dermatitis and otitis externa in animals, most frequently in dogs (12). The composition of the skin mycobiome can vary under pathological conditions and some species of *Malassezia* such as *M. sympodialis* and *Malassezia furfur* are found to be enriched in certain skin disorders (10). To date, a causative link between *Malassezia* and disease development has only been made for Pityriasis versicolor, while the role of the yeast in other pathologies remains correlative (10, 13, 14). Changes in the degree of colonization in diseased compared to healthy skin have been documented in dogs (15) but remain uncertain in humans (16).

**Figure 1:**
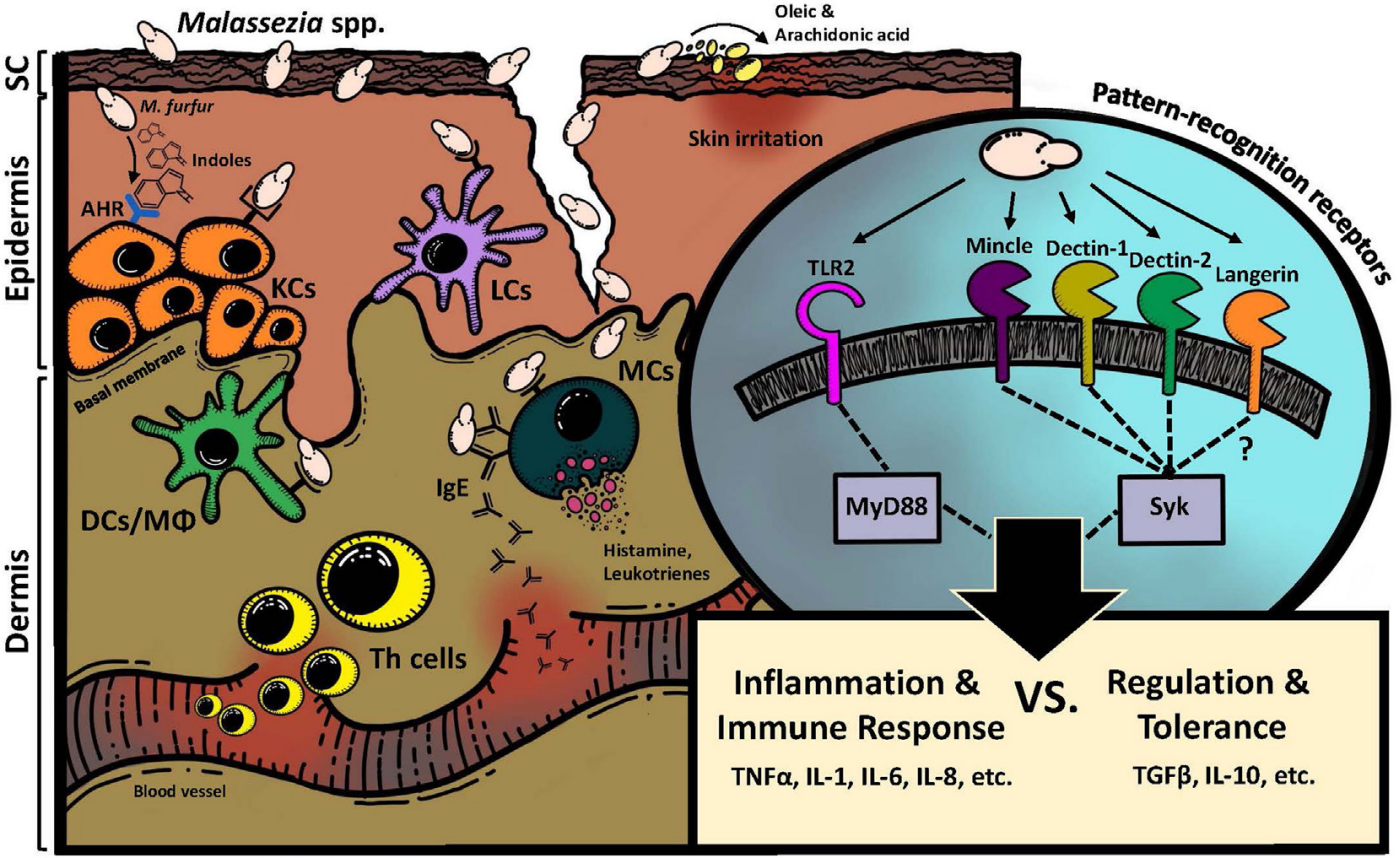
Interaction of *Malassezia* spp. with the mammalian skin. Direct interactions involve various PRRs, which recognize fungal cell wall constituents and are distinctly expressed on the surface of non-hematopoietic (i.e., keratinocytes) and hematopoietic cells (i.e., Langerhans cells, mast cells) of the skin. Spatial and temporal signal integration of different PRR signals results in the induction of inflammation and immunity or, alternatively, in the regulation and tolerance of the host toward *Malassezia* spp. Indirect interactions of *Malassezia* spp. with the skin include fungus-derived metabolites such as irritant fatty acids on the one hand and indoles that are potent agonists for the AhR, which is expressed by various skin cells, on the other hand. KCs, keratinocytes; LCs, Langerhans cells; DCs, dendritic cells; Mϕ, macrophages; MCs, mast cells; SC, stratum corneum; AhR, aryl hydrocarbon receptor.

The pathophysiology of *Malassezia*-associated skin conditions is largely unknown. The lack of knowledge on the cellular and molecular interactions between *Malassezia* spp. and the host preclude a better understanding of the factors determining commensalism versus disease. Herein, we review the current knowledge with regard to how the host recognizes *Malassezia* spp. and responds to it (Figure 1).

## Sensing of *Malassezia* spp. by the Host

Through their localization in the skin, *Malassezia* spp. interact primarily with keratinocytes, tissue-resident dendritic cells (DCs), and macrophages, as well as with myeloid cells that are recruited to the skin under inflammatory conditions. Activation of DCs is key for induction of adaptive immunity and memory formation. The fungus is recognized by the host either directly through interaction of fungal cell wall components with membrane bound pattern recognition receptors (PRRs) or indirectly through soluble metabolites that are released by *Malassezia* spp. The set of receptors expressed by the hematopoietic and the non-hematopoietic compartment are largely distinct.

### Direct Recognition of *Malassezia* spp. by Surface-Bound Receptors

The fungal cell wall is rich in carbohydrates and glycoproteins that are recognized by PRRs of the family of Syk-coupled C-type lectin receptor (CLR), which are expressed primarily by myeloid cells (17, 18). Binding to these receptors results in ligand internalization and activation of multiple signaling pathways, including the MAPK, NF-κB, and NFAT pathways as well as the inflammasome.

The polysaccharides of the *Malassezia* cell wall are organized differently than in other fungal species analyzed to date (19, 20). Moreover, the cell wall is surrounded by a lipid-rich outer layer (21). Several CLRs have been shown to respond to *Malassezia* spp. *in vitro*. The two FcRγ-associated receptors Dectin-2 and Mincle sense *Malassezia* spp., albeit through recognition of distinct ligands (22). While Mincle binds to two distinct glycolipids in *Malassezia*, Dectin-2 recognizes the fungus through α-1,2-linked mannose. High-mannose binding is a general feature of Dectin-2, which is reported to recognize a variety of fungi, including *Candida albicans, Saccharomyces cerevisiae, Blastomyces dermatitidis, Aspergillus fumigatus, Cryptococcus neoformans*, and *Fonsecaea pedrosoi* (23). In contrast, *Malassezia* spp. were initially found to be unique agonists of Mincle when a large panel of 50 different fungi was tested in a glycoconjugate microarray (24). More recently, other fungi such as *Pneumocystis carinii, F. pedrosoi*, and *Fonsecaea monomorpha* were also reported to engage Mincle (25–27), in addition to bacterial ligands (28–32), mammalian alarmins released from damaged cells (33, 34) and even cholesterol crystals (35, 36). Mincle is thus a highly pleiotropic receptor, which can bind chemically and structurally distinct ligands through at least two complementary binding sites (37–40). The β-glucan receptor Dectin-1, which was the first member of the family of Syk-coupled CLRs to be identified (41), was also found to sense *Malassezia* and was linked to the activation of the NLRP3 inflammasome (42). Finally, Langerin was suggested to act as a receptor for *Malassezia* in the skin due to its prominent expression by epidermal Langerhans cells and by a subset of dermal DCs. Direct binding of the fungus to recombinant Langerin was indeed observed (43, 44).

Activation of myeloid cells by *Malassezia* spp. *via* these different CLRs was shown to induce the secretion of proinflammatory cytokines. However, the relative contribution of individual receptors to fungal control *in vivo* during commensalism and in infectious settings remains to be determined. At least partial redundancy of receptors that signal *via* the same pathway may occur, similarly to what was found for other fungi (45, 46). Dissecting the role of Mincle in the context of *Malassezia* spp. in more detail will also be interesting in light of its reported antagonizing activity, e.g., in response to *Fonsacaea* spp. (27), and thus this receptor may also mediate regulatory or inhibitory responses to *Malassezia* spp.

In addition to CLRs, Toll-like receptors (TLRs), and in particular TLR2, also contribute to fungal recognition by the host. TLR2 was implicated in sensing of *Malassezia* spp. and inducing a proinflammatory response characterized by the release of cytokines, chemokines and antimicrobial peptides by keratinocytes (47–50).

The proinflammatory response is generally enhanced by lipid removal from the yeast to enhance exposure of fungal cell wall carbohydrates (51, 52). In contrast, thymic stromal lymphopoietin secretion from keratinocytes was found to be induced specifically by the lipid layer components of *M. restricta* and *M. globosa* but not by yeasts that were depleted of lipids (53).

### Indirect Interaction

Specific products of *Malassezia* metabolic pathways are thought to act as virulence factors promoting inflammation and pathology, while others downregulate the production of inflammatory mediators and thereby contribute to immune regulation. Fungal strains with altered production of such factors have been linked to *Malassezia*-associated skin disorders (54–56).

*Malassezia*-derived lipases and phospholipases, which are required to assimilate host-derived lipids, can initiate an inflammatory response in the skin by releasing unsaturated free fatty acids from the sebum lipids (57–60). Oleic acid has irritant and desquamative effects on keratinocytes (61–63), whereas arachidonic acid produces proinflammatory eicosanoids and leads to inflammation and damage to the stratum corneum, thereby contributing to the disruption of the epithelial barrier function and induction of abnormal keratinization (64).

*Malassezia furfur* is able to convert tryptophan into a variety of indole alkaloids. This pathway is mainly active if tryptophan is the sole source of nitrogen (65). *M. furfur*-derived indoles including malassezin, indirubin, and indolo [3,2-b] carbazole (ICZ) serve as potent ligands for the host aryl hydrocarbon receptor (AhR) and thereby potentially modify the function of all cells in the epidermis expressing this receptor (54, 55, 66, 67). For example, some tryptophan metabolites can promote apoptosis of melanocytes (68) or inhibit the respiratory burst in neutrophils (69). Given the broad spectrum of biological responses that are influenced by AhR activity, *M. furfur* may engage this pathway to modulate inflammation and/or promote skin immune homeostasis (70) but may also promote skin pathology (71) or even contribute to carcinogenesis (72). The significance of yeast-derived indoles in each of these contexts remains to be demonstrated *in vivo*.

## Innate Immunity to *Malassezia* spp.

The majority of what is currently known about the host response to *Malassezia* spp. is based on *in vitro* studies with isolated myeloid cells or keratinocyte cell lines. Stimulation of these cells with *Malassezia* yeast leads to the induction of mainly proinflammatory cytokines, chemokines, and antimicrobial peptides (22, 24, 47–52, 73–76). In line with an inflammatory character of the innate response to the fungus, the intraperitoneal injection of *Malassezia* into mice results in the recruitment of neutrophils to the peritoneum (24). Only few studies have examined regulatory cytokines such as IL-10 and TGF-β by the yeast (24, 49, 51, 74, 77), but these may be relevant with regard to the role of *Malassezia* spp. as a skin commensal.

Given the association of *Malassezia* spp. with inflammatory skin disorders and allergic responses, the fungus may also interact with mast cells. Progenitor cell-derived mast cells from atopic patients show increased release of proinflammatory cytokines upon stimulation with *Malassezia* (76) and are enriched in the skin of atopic eczema patients where they are positioned in the superficial dermis and can interact with the fungus (78). Mast cell activation in response to *Malassezia* spp. has also been reported in studies with bone-marrow-derived mast cells. These cells are directly activated by the fungus in a TLR2-dependent manner and release inflammatory mediators and cytokines (79). Moreover, the crosslinking of the high-affinity IgE receptor (FcεRI) by antigen-bound IgE can induce mast cell degranulation (79). Therefore, mast cells may contribute to further barrier disruption and thereby amplify the inflammatory response.

The access of *Malassezia* to immune cells in the skin may be facilitated by disruption of the epithelial barrier as it frequently occurs during chronic inflammation. Moreover, *Malassezia* spp. were reported to release nanovesicles/exosomes that contain immunogenic proteins and trigger increased release of cytokines by DCs (80).

## Adaptive Immunity to *Malassezia* spp.

As a commensal, *Malassezia* interacts continuously with the immune system. Therefore, cellular and humoral immune memory to the fungus can be evidenced in healthy individuals (81). Although there are fewer studies related to dogs when compared with humans, dogs also develop cellular and humoral immune responses to their commensal yeast, *M. pachydermatis* (82–84). Generally, the adaptive immune responses are heightened and qualitatively distinct in patients with *Malassezia*-associated diseases.

### Humoral Responses

During steady state, *Malassezia*-specific antibodies are predominantly of the IgG and IgM isotypes (81). In contrast, although *Malassezia*-specific IgE is not usually detected in healthy individuals, it is common in atopic patients (85). A positive correlation was found between the sensitization to *Malassezia*-specific IgE and the severity of atopic dermatitis (86, 87). Similar observations were made in atopic dogs (83, 84). However, whether the IgE response plays a pathogenic role in atopic and other *Malassezia*-associated inflammatory disorders or rather serves as a marker for the severity of disease remains unclear.

### T Cell Responses

Patients with atopic dermatitis often show positive skin prick test and atopic patch test reactions to *Malassezia* (85). T cell-responsiveness to *Malassezia* in such patients was associated with a Th2 response (88), in line with the classical paradigm of Th2-polarized allergic T cells. GATA3^+^ T cells were identified in pityriasis versicolor lesions (89) and likewise *Malassezia*-specific T cell in allergic dogs were found to be strongly polarized toward a type 2 response (82). More recently, other T helper cell subsets such as Th17 and Th22 cells have been found enriched in allergic individuals (90, 91) as well as in non-allergic immune-mediated skin diseases such as psoriasis (92). Consistent with this notion, *Malassezia*-reactive skin homing T cells from *Malassezia*-sensitized atopic dermatitis patients comprise not only Th1 and Th2 subsets but also IL-17- and IL-22-secreting cells (93). Of note, IL-4/IL-17 coproducers have also been described in the context of atopic eczema especially in children (94). Importantly, Th17 differentiation is a hallmark of T cell responses induced by CLR signaling (46) and T cells directed against other fungi, in particular *Candida* spp., belong predominantly to the Th17 subset (95). Whether and how IL-17 and/or IL-22 may contribute to pathogenicity in atopic dermatitis remains to be determined. It is also unknown to which subset *Malassezia*-specific T cells belong in healthy individuals and to what extent T cell plasticity contributes to sensitization.

### *Malassezia* Allergens

To date, 13 *Malassezia*-derived allergens have been identified from *M. furfur* and *M. sympodialis* (3, 96). Interestingly, more allergens are released from *M. sympodialis* when cultured at the increased pH conditions of atopic skin compared with culture at the pH of healthy skin (97). Several of the known allergens belong to a class of phylogenetically highly conserved proteins and display a high degree of homology with the corresponding mammalian proteins. Cross-reactivity between *Malassezia*-derived allergens and endogenous human proteins (e.g., thioredoxin, manganese-dependent superoxide dismutase) has been indeed demonstrated (93, 98, 99). Therefore, the induction of autoreactive T cells by *Malassezia* allergens may play a role in sustained inflammation.

## Conclusion

*Malassezia* spp. have been implicated in various pathologies. Yet, direct evidence for a causal relationship between *Malassezia* spp. and the mammalian host remains elusive. For instance, it is unclear whether *Malassezia* actively promotes atopic dermatitis or whether the inflammatory environment in the atopic skin triggers a dysregulated immune response toward the fungus.

At the basis of this is the key question of what determines the balance between commensalism and pathogenicity of *Malassezia* spp. The answer likely relates to changes occurring in both the fungus (55) (e.g., variable secretion of AhR agonists) and in the host (e.g., barrier defects, changes in immune polarization) which are responsible for promoting the development of pathology. Changes in the environment such as seasonal variations in sebum production have also been linked to altered disease prevalence (100).

Inter-species variations in the skin mycobiome may further contribute as different species of *Malassezia* can induce variable inflammatory responses (51, 75, 101). Moreover, *Malassezia* spp. have been shown to display a large intra-species diversity (73) similarly to what is known for other opportunistic fungal pathogens (102), and thus the exact composition of *Malassezia* strains and species present in an individual at a given time may contribute to different outcomes in the interaction between the fungus and the host. The recently completed assembly and detailed annotation of the genome of *M. sympodialis* makes an important contribution to approach this complexity (103). Future research will help fill the important gaps in our knowledge on the pathophysiology of and the host response to *Malassezia in vivo*. Enhanced understanding of host-*Malassezia* interactions may contribute to improved diagnostic and therapeutic options for patients affected by *Malassezia*-associated pathologies.

## Author Contributions

Both authors listed have made a substantial, direct, and intellectual contribution to the work and approved it for publication.

## Conflict of Interest Statement

The authors declare that the research was conducted in the absence of any commercial or financial relationships that could be construed as a potential conflict of interest.
